# Aging-Related Tau Astrogliopathy in Aging and Neurodegeneration

**DOI:** 10.3390/brainsci11070927

**Published:** 2021-07-13

**Authors:** Heather McCann, Briony Durand, Claire E. Shepherd

**Affiliations:** 1 Neuroscience Research Australia, Barker Street, Sydney, NSW 2031, Australia; h.mccann@neura.edu.au (H.M.); b.durand@neura.edu.au (B.D.); 2 Department of Pathology, The University of New South Wales, Kensington, Sydney, NSW 2031, Australia

**Keywords:** astrocytes, aging-related tau astrogliopathy, tauopathy, tau propagation, aging, neurodegeneration

## Abstract

Astrocytes are of vital importance to neuronal function and the health of the central nervous system (CNS), and astrocytic dysfunction as a primary or secondary event may predispose to neurodegeneration. Until recently, the main astrocytic tauopathies were the frontotemporal lobar degeneration with tau (FTLD-tau) group of disorders; however, aging-related tau astrogliopathy (ARTAG) has now been defined. This condition is a self-describing neuropathology mainly found in individuals over 60 years of age. Astrocytic tau accumulates with a thorny or granular/fuzzy morphology and is commonly found in normal aging as well as coexisting with diverse neurodegenerative disorders. However, there are still many unknown factors associated with ARTAG, including the cause/s, the progression, and the nature of any clinical associations. In addition to FTLD-tau, ARTAG has recently been associated with chronic traumatic encephalopathy (CTE), where it has been proposed as a potential precursor to these conditions, with the different ARTAG morphological subtypes perhaps having separate etiologies. This is an emerging area of exciting research that encompasses complex neurobiological and clinicopathological investigation.

## 1. Introduction

Astrocytes play an essential role in maintaining cellular homeostasis in the central nervous system (CNS) and are active contributors to neuronal function [1]. Changes in astrocytic function occur during normal aging and astrocytic pathology underlies a large number of neurodegenerative disorders. Recently, aging-related tau astrogliopathy (ARTAG) has been used to describe tau pathology accumulating in astrocytes in the aged brain [2]. We are starting to understand its prevalence in aging and neurodegeneration, its clinical relevance, pathological spread and its underlying biochemical profile. This review highlights the current literature and knowledge gaps in this area.

## 2. Overview of Astrocyte Biology and Function

Astrocytes are stellate-shaped glial cells that have a variety of essential functions within the brain. They outnumber neurons approximately fivefold [3] and are arranged in highly organized territories so each individual astrocyte is responsible for a particular domain. Within these domains their long processes allow them to contact multiple structures including neurons, myelin, other glia and blood vessels, with an individual astrocyte estimated to contact hundreds of dendrites and hundreds of thousands of synapses in the human brain [4,5]. Although considered non-excitable due to the inability to generate action potentials, astrocytes express sodium and potassium channels to provide inward currents [6] and are able to regulate intracellular calcium to communicate with nearby neurons and glia [1]. Astrocytes are also able to communicate with neighboring cells via gap junctions formed by connexins 30 and 43 [7] to allow direct electrical and biochemical coupling [8]. Through these mechanisms, astrocytes are able to influence the excitation or inhibition of surrounding neuronal networks and shape neuronal activity [9]. Indeed, astrocyte function is critical for normal synaptic transmission through the trafficking and redistribution of neuroactive substances, such as glutamate [10].

Two main astrocyte types associated with human white matter and cortex were proposed in the early 20th century [11] and remain relevant today. Protoplasmic astrocytes are the most numerous and are found organized in layers II-VI of the cortex [12]. They consist of a dense network of processes which project to the surrounding vasculature to form the glia limitans and the outermost wall of the blood brain barrier (BBB) and are also closely associated with synapses [12]. Fibrous astrocytes contain fewer, straighter processes with more overlap and are found along white matter tracts where their processes connect them with the neurovascular unit [13]. However, various astrocytic subtypes have been identified based on their genetics, biochemistry, physiology, morphology and location [12,14,15] and an increasing pool of research supports the notion that there is considerable heterogeneity in astrocyte populations both between and within brain regions and neuron networks [16].

The multicellular networks formed by astrocytes are necessary to maintain normal CNS function, which include but are not limited to:

- Regulation of blood flow through release of molecules that can dilate blood vessels, such a nitric oxide, prostaglandin and arachidonic acid [17].
- Contribution to the neurovascular unit along with neurons and endothelial cells. The end feet of protoplasmic perivascular astrocytes form the most external layer of this unit, the glia limitans, [13,18] an important constituent of the BBB. It is thought that astrocytes act on endothelial cells to maintain this layer of protection to the central nervous system [19].
- Synapse function and activity, where astrocytes are prompted by calcium to release molecules such as glutamate, GABA and purines which in turn can alter neuronal excitability and enhance synaptic function by turnover of neurotransmitters [6,13,20,21].
- Maintenance of cellular homeostasis through aquaporin 4 (AQP4) water channels to regulate fluid homeostasis [22], and regulation of potassium, sodium and calcium ions to maintain pH [23,24].
- Repair after CNS damage including glial scar formation [1].
- Production of pro-and anti-inflammatory cytokines in response to infection and injury [25].

When astrocytic function is altered it can have widespread downstream effects and cause or contribute to disease. A direct example of this is seen in Alexander disease where gain of function mutations in glial fibrillary acidic protein (GFAP), which is the major intermediate filament (IF) expressed by astrocytes, causes leukodystrophy and neuronal loss [26]. GFAP is a type III IF that, along with other IFs, is involved in the structure and function of the astrocytic cytoskeleton and plays a role in cell communication, mitosis, blood brain barrier integrity and cellular repair [27]. GFAP upregulation is classically used as a marker of astrocyte activation, such as that seen following insult, injury or ischemia. This so-called reactive astrogliosis may be mild and involve temporary cellular hypertrophy and restoration of a normal cellular state or it may be more severe resulting in cellular proliferation and permanent remodelling of the cytoarchitecture through glial scar formation. Whilst normally reparative in function, astrocytes may cause further damage if their normal reactive response is impaired due to conditions such as degeneration or senescence [1]. 

Senescence describes an age-related loss of function, and astrocytes are known to express classic markers of senescence as they age, including p16 and p21 [28]. When senescent astrocytes are co-cultured with neurons there is reduced neuronal survival and altered synaptic function [29]. Stress has also been shown to induce premature senescence [30] indicating this phenotype can be reached via various means. Increased markers of astrocyte senescence have been identified in both normal ageing and in neurodegenerative disorders such as Alzheimer’s disease [31]. In vitro studies suggest that tau and amyloid-beta protein can switch a healthy astrocyte into one with a senescent phenotype [31,32] and Alzheimer’s disease patients show greater senescence than their age-matched counterparts, indicating that senescent astrocytes accumulate with normal aging and further increase with the onset of AD [31]. 

## 3. Tau and Tauopathies

Tau is a microtubule-associated protein encoded by the MAPT gene located on chromosome 17 [33]. The main role of tau is to maintain axonal transport by stabilizing microtubules through the process of phosphorylation and dephosphorylation [34]. In the adult human brain there are 16 exons and messenger RNA splicing of exons 2,3 and 10 produces six main tau isoforms referred to as 3 or 4 repeat tau, related to the number of microtubule binding domains it contains [35]. The ratio of these isoforms differs depending on life-stage with only 3R forms existing during development, while a 1:1 ratio of the isoforms exist in adulthood [35]. The more repeats, the better the microtubule binding and stabilizing capabilities, meaning that 4R tau has better stabilizing properties than 3R but is also more prone to aggregation [36,37]. Phosphorylation of tau is regulated by protein kinases, with altered regulation or activity of kinases and phosphatases thought to contribute to a hyperphosphorylated state [38,39]. In neurons, hyperphosphorylation of the tau protein disrupts the microtubule structure leading to the accumulation of tau aggregates within the cell soma [39,40] and the eventual formation of paired helical filaments [41,42] and neurofibrillary tangles [43]. Post-translational modification of the tau protein such as acetylation, glycosylation, methylation, nitration truncation and ubiquitination can also lead to pathological aggregation in both neurons and glial cells [38,44,45,46,47], although tau acetylation is known to be rarer in astrocytes [48]. Indeed, glial tau is ultrastructurally similar to neuronal inclusions [49] and pathology shows equivalent tau stages to neuronal pretangles and neurofibrillary tangles [46]. 

The term tauopathy denotes a range of neurodegenerative diseases where atypical accumulation of tau protein is found in neurons and glial cells [50]– see Table 1 showing common sporadic neurodegenerative tauopathies, their characteristic tau isoforms and affected cell populations. Familial MAPT diseases also fit within this spectrum of disease but will not be discussed in any detail in this review. These sporadic disease groups can be further broken down into those with predominantly neuronal and/or astrocytic tau inclusions. 

In Alzheimer’s disease (AD) and primary age-related tauopathy (PART) the tau pathology is mostly neuronal, however both astrocytic and neuronal pathology is characteristically seen in FTLD-tau [46] and CTE [51]. In FTLD-tau, pathology occurs in neurons, oligodendroglia and astrocytes, predominantly as 4R tau protein aggregates in progressive supranuclear palsy (PSP), corticobasal degeneration (CBD), globular glial tauopathy (GGT) and argyrophilic grain disease (AGD) and 3R tau aggregates in Pick’s disease (PiD) [52,53]. However, exceptions to this rule exist as immunohistochemical investigations using different tau antibodies have shown that some tufted astrocytes in PSP contain 3R tau and some astroctyes in PiD contain 4R tau. In addition, astrocytic tau phosphorylation sites, conformational modifications, truncation and ubiquitination was shown to differ between FTLD-tau subtypes with more modifications in cases with MAPT mutations and GGT compared to PSP, CBD and AGD. Furthermore, not all astrocytes contained the same tau modifications, regardless of the FTLD-tau subtype [46], demonstrating the complexities of astrocytic heterogeneity [54].

**Figure 1:**
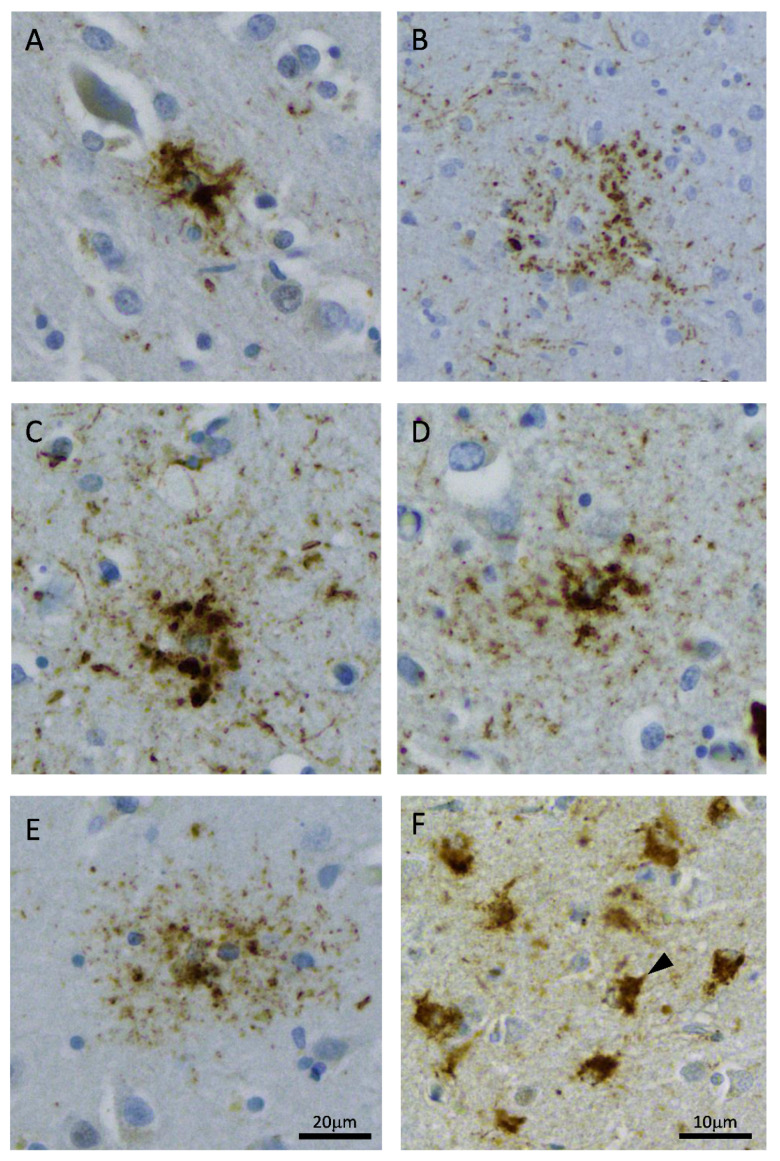
Astrocytic morphologies of frontotemporal lobar degeneration with tau (FTLD-tau) and aging-related tau astrogliopathy (ARTAG), immunostained with AT8-tau antibody. Dense tau fibrils in a tufted astrocyte in progressive supranuclear palsy (PSP, **A**); annular punctate tau in an astrocytic plaque in corticobasal degeneration (CBD, **B**); a globular astroglial inclusion in globular glial tauopathy (GGT, **C**); short, thick tau processes in a ramified astrocyte in Pick’s disease (PiD, **D**); ramified and bushy tau process in a granular/fuzzy astrocyte (**E**) and short dense tau deposits in thorny astroctyes (arrowhead) (**F**). Granular/fuzzy (**E**) and thorny (**F**) astrocytes are pathogno-monic of argyrophilic grain disease (AGD) and ARTAG. (**A**–**E**) images at 200× magnification and (**F**) at 400× magnification.

In addition to the biochemical variations in astrocytic tau, morphological variations are an important classification tool to differentiate between FTLD-tau subtypes [50,55]—see Table 2 and Figure 1. 

FTLD-tau subtypes are further distinguished by the differing anatomical location of their astrocytic tau aggregates. In PSP and CBD, pathology concentrates in the cortex and basal ganglia [60,61], in AGD pathology is in limbic and temporal regions [62], in GGT the grey matter and in PiD pathology is concentrated in cortex and limbic regions [61].

A further differentiating feature of FTLD-tau astrocytes is that particular morphologies may correspond to astrocyte subtypes. Astrocytic plaques, tufted astrocytes and ramified astrocytes of PiD are predominantly found in grey matter and are therefore believed to be protoplasmic whereas thorny astrocytes are thought to occur more commonly in fibrous astrocytes due to their morphology and frequent white matter location [49,63,64]. Although convenient, this current classification of astrocytes into protoplasmic and fibrous subtypes is considered by many to be too simplistic. The suspected complexity of astrocytic subtypes raises questions whether different subtypes may show selective vulnerability for certain tau modifications or different tau species may modify the morphology of the astrocyte.

The final astrocytic tauopathy to be discussed is the focus of this review, the recently classified ARTAG. Despite it being a relatively new neuropathological entity, the ability to be able to reliably identify ARTAG and differentiate it from other astroglial tauopathies has proven relatively successful [2]. ARTAG is a 4R tau disorder defined by the presence of hyperphosphorylated tau in two distinct morphologies, thorn-shaped astrocytes (TSAs) and granular/fuzzy astrocytes (GFAs)– see Figure 1. The most common structures are TSAs, which are also seen in AGD and have short “thorny” processes which accumulate in the subpial, subependymal, perivascular, white matter and less frequently the grey matter of medial temporal lobe, cortex and underlying white matter, subcortex and brainstem structures. Specifically, the amygdala is thought to be a predilection site for all ARTAG types [65]. The criteria for classifying ARTAG is based on these locations, anatomical distributions and severity of pathology [2]. TSAs are proposed to affect fibrous astrocytes (or more specifically, interlaminar astrocytes) due to their predominant white matter location and their predilection for the glia limitans [2]. They have a similar pattern of tau phosphorylation to TSAs in AGD [46] and lack truncated tau [66]. The GFAs seen in ARTAG are also not biochemically or morphologically distinct from those seen in AGD, are found predominantly in the grey matter and have bushy processes with a granular distribution of phosphorylated tau throughout the processes and perinuclear staining. They appear to affect protoplasmic astrocytes in the grey matter, stain with diverse tau antibodies and display some argyrophilia [67,68], suggesting only minor tau modifications are present. Both structures may coexist and it is unknown whether they reflect two distinct pathogenic mechanisms. 

## 4. ARTAG in Aging and Non-Astrocytic Tauopathies

ARTAG is associated with advancing age, particularly in those over 60 years old [2] and is seen more often in males [65]. Studies have consistently shown that ARTAG is present in more than a third of elderly cases, including a large post-mortem study of community based elderly individuals [69] and smaller studies reporting ARTAG in 25–50% of neurologically normal cases [65,70] and in 100% of a small sample of centenarians over the age of 110 years [71]. Similarly, investigations of non-tauopathy neurodegenerative disease cohorts have also found the presence of ARTAG with differing prevalence, although always increasing with age and co-pathologies, including cerebrovascular disease [65,70,72]. A 25–56% prevalence of ARTAG has been recorded in synucleinopathies [65,70,73], 15% in sporadic and genetic Creutzfeldt-Jakob disease (CJD) [74], 40% in motor neuron disease [65] and in 43% of a small Huntington’s disease cohort [75]. These studies have determined that TSAs are usually the most common astrocyte morphology and are distributed through cortical, medial temporal lobe, subcortical and basal forebrain structures in subpial, subependymal, white matter and perivascular locations [65,69,70,72,73,74,75,76]. The amygdala has been identified as a predilection site for ARTAG in aging, and AD pathology (including PART) has been associated with subpial, white matter and perivascular ARTAG in the limbic region of up to 64% of AD and PART cases [65,70,77,78,79]. However, the distribution of GFAs in PART has been found to be more similar to the distribution seen in AGD than AD [67] and although white matter TSAs have been associated with increasing Braak stage [77], not all studies support this association [80]. However, its presence, distribution and milder severity in non-tauopathies suggests that ARTAG may be an independent disease process [74,75] more likely to be a non-specific process of ageing [75] but that it could also be expedited by coexisting disease processes [74,75].

The significance of ARTAG astrocyte morphology and location are not entirely clear, however, there are several proposed mechanisms that are subject to further enquiry. Subpial and perivascular ARTAG have been proposed to be associated with significant BBB dysfunction due to the proximity to the glia limitans [65], and BBB disruption is more common with advancing age [81]. Indeed, connexin-43, which is expressed by astrocytes at the BBB and plays a major role in both gap junction and immune functions [82] is increased 6-fold in ARTAG and there was also a significant increase in AQP4, indicating BBB dysfunction and local hypoperfusion [83]. As BBB permeability has been proposed as an early mechanism underlying disease, it has been suggested that ARTAG may be an indicator of an early neurodegenerative processes [83]. The relationship between astrocyte senescence and neurodegeneration is also under investigation, with in vitro cell culture studies suggesting that astrocyte senescence leads to neurodegeneration, making astrocytic tau accumulation and senescence possible targets for therapeutic intervention [32]. Immunohistochemical experiments have shown that TSAs display an immunoreactive profile consistent with senescence, including reduced GFAP, vimentin and YKL-40 and increased SOD2 immunoreactivity [66]. Further understanding of the potentially varied ARTAG cellular phenotype and the regional distribution might provide some clues to discriminate the etiology.

Whether ARTAG is associated with a clinical phenotype is not clear as the lack of available pre-mortem clinical data has made it difficult to draw any meaningful clinicopathological conclusions. ARTAG is frequently present with AD pathology [65] and to date most studies assessing the clinical relevance of ARTAG have been in cohorts with significant AD pathology. Not surprisingly, results in cases with coexisting pathology have been conflicting with some studies showing no association between ARTAG and cognitive status [72,77] and others demonstrating relationships with an aphasic syndrome in AD [84], worsening language and visuospatial function [79] and cognitive decline with or without Parkinsonism [68]. The regional distribution and contribution of multiple pathologies must also be considered as a large longitudinal investigation of those over 90 years old demonstrated that cortical ARTAG, hippocampal sclerosis and cerebrovascular disease were associated with dementia but limbic and brainstem ARTAG were not [85]. These studies highlight the importance of considering ARTAG type and location when validating findings and interpreting data.

## 5. ARTAG in Other Astrocytic Tauopathies

Similar to the non-astrocytic tauopathies and other neurodegenerative disorders, ARTAG has been observed frequently in the astrocytic tauopathies, specifically in FTLD-tau.The prevalence of ARTAG in primary FTLD-tau is high and has been reported in up to 100% of PSP, CBD and AGD cases although there appears to be a lower incidence of ARTAG in PiD [65,78]. Whilst this may be partially explained by the predominantly 3R nature of PiD, the frequency of ARTAG was still higher in PiD than in AD and PART where both 3R and 4R tau isoforms dominate [78]. However, the pattern of ARTAG in the grey matter has been shown to correspond to the independent patterns of pathologies in PSP, PiD and CBD and the presence of TSAs and GFAs adjacent to one another in the grey matter of FTLD-tau cases have been speculated to represent various stages of astrocytic maturation from GFAs to disease-specific astrocytic plaques and ramified astrocytes [65]. In addition, astrocytes resembling GFAs and globular-like astrocytes in primary FTLD-tau are found in grey matter regions that display little neuronal pathology, indicating ARTAG may be a precursor to both neuronal and other glial tauopathies [65,78,83]. The possibility of tau seeding as a means of sequential tau pathology spread will be discussed later in this review. 

**Figure 2:**
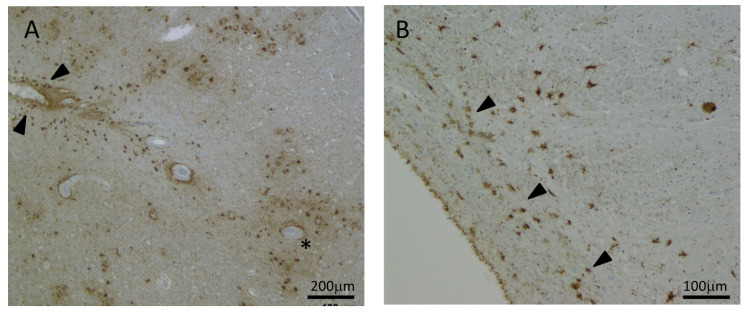
Thorn-shaped astrocytes in subpial (arrowheads) and perivascular sulcal depth (asterisk) arrangements in chronic traumatic encephalopathy (CTE), 50× magnification (**A**); subpial thorn-shaped astrocytes (arrowheads) adjacent to the substantia nigra region of the midbrain, 100× magnification (**B**). Immunostaining with AT8-tau antibody.

Along with neuronal inclusions, astrocyte pathology indistinguishable from TSAs is a consistent neuropathological feature of chronic traumatic encephalopathy (CTE) [51]. The pathology is primarily located in a perivascular arrangement in sulcal depths but also in other regions of cortex, white matter, subcortical, brainstem and cerebellar regions [51,86]—see Figure 2. Tau pathology found in TSAs in both ARTAG and CTE are both composed of the 4R isoform and are phosphorylated at similar residues and lack staining against tau-C3 [87]. These similarities highlight the difficulties in differentiating the astrocytic pathology in CTE from ARTAG [69] and go some way to explaining why a concomitant diagnosis of CTE and ARTAG is not recommended [51]. However, there is emerging evidence that astroglial tau may be the most important neuropathologic change seen in CTE, whilst the neuronal tau found in sulcal depths is more closely associated with AD neuropathologic change [88]. Indeed, TSAs are more commonly found accumulated at the sulcal depths in a perivascular distribution in CTE cases [88] and this has been corroborated by our unpublished findings in cases of CTE at the Sydney Brain Bank (manuscript in preparation). It has also recently been suggested that TSAs might be related to trauma while GFAs relate to neurodegeneration. Indeed, during a small case study of two individuals with large arachnoid cysts it was found that TSA pathology had resulted from long-term mechanical stress, and it is proposed that these inclusions may be different from neurodegenerative-associated ARTAG in the form of GFAs [89]. Further investigation in larger cohorts of cases of mechanical stress would be needed to answer this question. 

Interestingly, recent studies have demonstrated that striatal aneuploid astrocytes are capable of differentiating into neurons and forming neuronal circuits following ischemic brain injury [90]. We, and others, have demonstrated aneuploidy in both neurons and glia in various neurodegenerative disorders, including AD and Lewy body disease (for review see [91]) and more recent studies have demonstrated significant glial aneuploidy in FTLD-tau [92]. Transgenic mouse studies also demonstrate chromosome misegregation and aneuploidy in cells expressing mutant tau protein [92] indicating a possible link between these pathological processes. Senescence may also exacerbate these pathologies as both neuronal and non-neuronal aneuploidy has been shown to increase with age [91]. Aneuploidy may represent a missing link between neuronal and astrocytic tau pathologies, which would be important to explore given the high prevalence of ARTAG in FTLD-tau and CTE.

## 6. Astrocytic Tau Propagation

**Figure 3:**
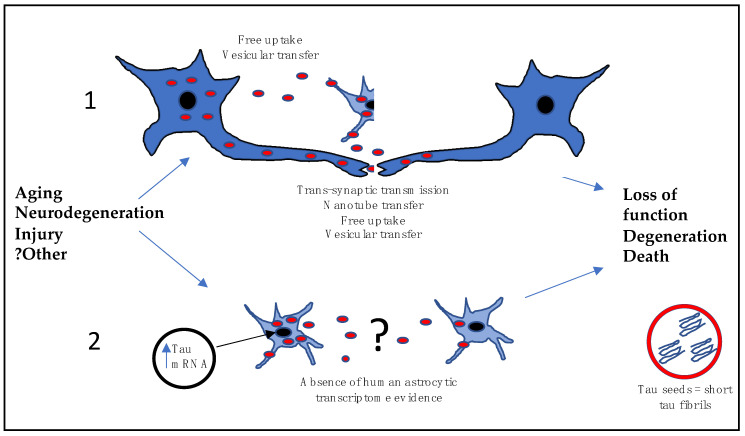
Schematic of two astrocytic tau propagation hypotheses. Small tau fibrils or “seeds” (red dots) may be formed in the cytoplasm of neurons or astrocytes after release from endosomal vesicles. Dissemination may occur from neuron-to-neuron by mechanisms such as trans-synaptic transmission, free uptake or vesicular or nanotube transfer to neighboring cells, while neuron-to-astrocyte and astrocyte-to-astrocyte transmission is not clear and may occur via trans-synaptic transmission, vesicular uptake or free transfer. Hypothesis 1 relies on neuronal release of tau, which is taken up by nearby astrocytes. Hypothesis 2 sees astrocytes upregulate tau and kinase expression leading to tau hyperphosphorylation and spread to other cells, however there is currently little evidence to support this.

While the exact mechanism of astrocytic tau pathology has not been fully elucidated, it is theorized that tau spreads through the brain via the release of small tau inclusions or “tau seeds”. These seeds are thought to consist of short tau fibrils which disseminate from cell to cell where they begin to accumulate [93,94]. The mode of dissemination is still poorly understood, but may be the result of release from endosomal vesicles into the cytoplasm, where tau fibrils are formed, followed by trans-synaptic transmission, free uptake or vesicular or nanotube transfer to neighboring cells [93]. The end result is loss of function and degeneration of the tau-containing cells—see Figure 3 [32]. In support of this hypothesis, astrocytic tau propagation studies have shown that human brain homogenates from AGD, PSP and CBD cases were able to produce similar lesions in mice transgenic for wild-type human tau and that these lesions spread in a similar pattern to the human condition [95]. Importantly, homogenates from human ARTAG cases containing TSAs only and inoculated into wild-type mice have been able to produce tau propagation in astrocytes, oligodendrocytes, neurons and white matter fibres, showing that astrocytes are highly involved in tau propagation [66]. Further work by the same group showed that human ARTAG homogenates propagated tau in wild-type mice, but mostly in neurons and oligodendrocytes [96], and recent studies have demonstrated that astrocytic pathology does not propagate in the absence of neuronal tau [25,97], suggesting there are other propagation factors at play. Perhaps clues may be derived from a cell culture propagation model, where human PiD brain extracts were used to infect HEK293T cells expressing 3R tau and extracts from AGD, CBD, and PSP human samples were transmitted to HEK293 cells expressing 4R tau. The results demonstrated that tau propagation in HEK cells required pairing of tau isoforms between the inoculum and the recipient substrate, meaning that propagation only occurs between cells expressing like-for-like tau [98]. Thus, there are currently two propagation hypotheses for ARTAG development, the first where neurons release tau which is taken up by nearby astrocytes and the second where astrocytes upregulate tau expression and kinases which lead to hyperphosphorylation and spread to other cells- see Figure 3 [89]. Whilst there is convincing support from cellular and animal models for the former hypothesis, this mode of propagation is not supported in disorders such as PiD where 4R ARTAG exists in association with 3R neuronal and astrocyte inclusions [78]. Despite this, the first tau propagation hypothesis is favored due to the fact that mRNA studies have failed to demonstrate significant expression of tau in astrocytes [99,100], suggesting they are unable to initiate this pathology in the absence of neuronal tau. However, there is a notable absence of cell-specific, tau transcriptomic studies carried out using human tissue. 

## 7. Staging of ARTAG

A clear staging classification system helps provide a better understanding of the development of pathology and disease. However, unlike neuronal tau pathology, identifying a unified staging system for ARTAG has proven difficult and is likely due to the differences in ARTAG morphologies, regional and disease-specific variations and the potential differences in etiology. Nevertheless, complex staging systems have been proposed which outline patterns for ARTAG [78]. These staging systems are based on the following:

- ARTAG location- subpial, white matter and grey matter (subependymal ARTAG does not have a distinct pattern).
- ARTAG subtype- GFA versus TSA.
- ARTAG association with FTLD-tau disorders- PSP, CBD, PiD.

**Figure 4:**
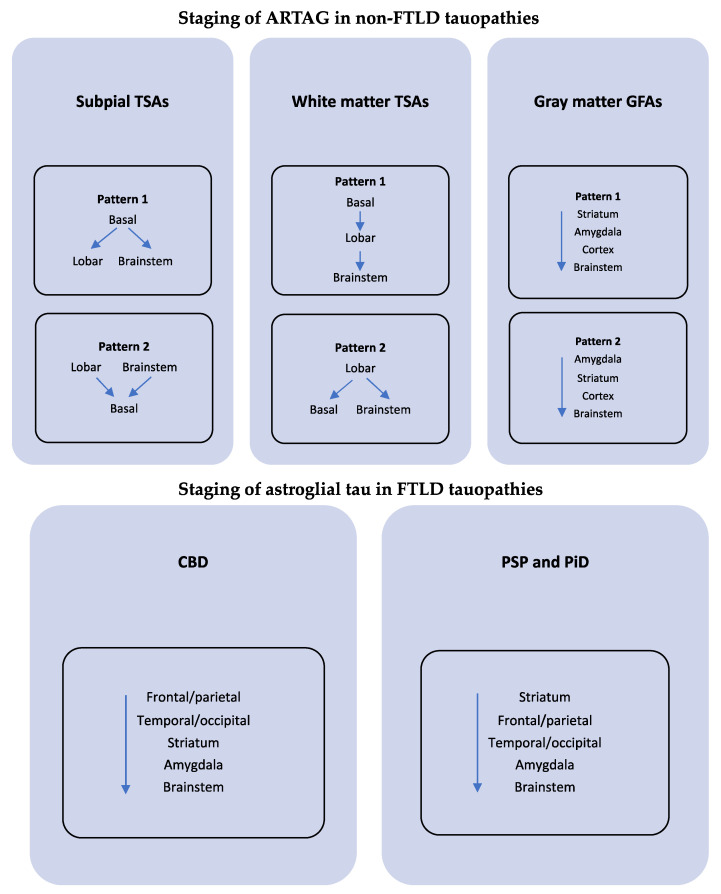
A simplified schematic for aging-related tau astrogliopathy (ARTAG) staging systems based on Kovacs et al. Acta Neuropathol Commun. 2018. Staging is classified according to ARTAG location- subpial, white matter, subependymal thorn-shaped astrocytes (TSAs) and grey matter granular/fuzzy astrocytes (GFAs) in non-frontotemporal lobar degeneration tauopathies (FTLD-tau). No distinct pattern has been identified for subependymal ARTAG. Multiple patterns of spread have been proposed for all classifications (**top** panel). Basal regions = basal forebrain and amygdala; lobar regions = frontal, parietal, temporal and occipital lobes.

Multiple patterns of spread have been proposed for each of the classifications listed above that have been more comprehensively explained elsewhere [65,78]. However, the general and overarching findings suggest that the origins of GFAs in the grey matter are distinct from TSAs in subpial, subependymal, white matter and perivascular locations and these pathologies should be distinguished- see Figure 4. Furthermore, the location of the TSAs is important, as white matter TSAs are not associated with subpial TSAs in lobar regions, but these ARTAG types are strongly associated in basal forebrain and brainstem regions [78]. These findings support distinct etiologies for ARTAG types and differing patterns of spread depending on the initial cause, type and location of pathology [78]. It is not yet known how ARTAG in CTE fits into this framework and if common sequential patterns can be discerned in cases with distinct etiologies. Given there are many etiologies and pathways of ARTAG spread, a single hierarchical progression of this currently unified pathology is unlikely.

Staging schemes proposed for both ARTAG and pathognomonic astroglial tau in the FTLD-tau disorders corticobasal degeneration (CBD), progressive supranuclear palsy (PSP) and Pick’s disease (PiD, bottom panel). It is suggested that the etiology of grey matter GFAs are distinct from TSAs and as such these pathologies should be distinguished.

## 8. Concluding Remarks

The importance of normal astrocytic function within the central nervous system cannot be understated. Remarkably, even up to 24 h postmortem, astrocytes and microglia increase their gene expression and alter their morphology in an attempt to rescue degenerating neurons [101], indicating that their resilience and response programming are crucial and continue to function even after death. 

Over the last 20 years, our focus on astrocytic dysfunction and pathology has enabled significant progress in an area of research that holds great promise for the treatment of a number of brain disorders. The identification of ARTAG in 2016 [2] marks a significant milestone in this journey and there is now good conceptual evidence for a link between ARTAG pathology, particularly GFAs, as a precursor to FTLD-tau and accumulating evidence of the importance of TSAs in the pathogenesis of CTE [88,102,103]. Whilst encouraging, there are still many knowledge gaps and further research is required to answer a number of fundamental questions pertaining to the etiology of ARTAG, its clinical significance and its role in neurodegeneration. Answering these questions will only be possible through a greater understanding of the complex, functional relationship between astrocytes and neurons and their associated pathologies in the context of the significant heterogeneity that exists at a regional, cellular and molecular level in the human brain. 

## Figures and Tables

**Table 1:** Common 3R and 4R tauopathies.

**3R and 4R tauopathies**
*Neuronal* Alzheimer’s disease Primary age-related tauopathy *Neuronal and astrocytic* Chronic traumatic encephalopathy (note astrocytic tau is 4R only)
**4R tauopathies**
*Neuronal and astrocytic* Progressive supranuclear palsy Corticobasal degeneration Globular glial tauopathy Argyrophilic grain disease *Astrocytic* Aging-related tau astrogliopathy
**3R tauopathy**
*Neuronal and astrocytic* (note astrocytic tau is predominantly 4R) Pick’s disease

**Table 2:** FTLD-tau astrocytic morphology.

FTLD-Tau Subtype	Astrocyte Morphology	Tau Distribution within Astrocyte
Progressive supranuclear palsy	Tufted astrocyte	Dense fibrils forming tufts stretching outward from the nucleus [56]
Corticobasal degeneration	Astrocytic plaque	Irregular annular structures with punctate tau located in distal processes [57]
Globular glial tauopathy	Globular astrocytic inclusions	Globules and granules in proximal processes [50]
Argyrophilic grain disease	Granular/fuzzy astrocytes, thorny-shaped astrocytes	Ramified bushy processes (granular/fuzzy) and short dense perinuclear deposits (thorny) [58]
Pick’s disease	Ramified astrocytes	Thick tau positive processes [59]

## Data Availability

Not applicable.
